# Extracellular Vesicles Regulate Biofilm Formation and Yeast-to-Hypha Differentiation in Candida albicans

**DOI:** 10.1128/mbio.00301-22

**Published:** 2022-04-14

**Authors:** Leandro Honorato, Joana Feital Demetrio de Araujo, Cameron C. Ellis, Alicia Corbellini Piffer, Yan Pereira, Susana Frases, Glauber Ribeiro de Sousa Araújo, Bruno Pontes, Maria Tays Mendes, Marcos Dias Pereira, Allan J. Guimarães, Natalia Martins da Silva, Gabriele Vargas, Luna Joffe, Maurizio Del Poeta, Joshua D. Nosanchuk, Daniel Zamith-Miranda, Flávia Coelho Garcia dos Reis, Haroldo Cesar de Oliveira, Marcio L. Rodrigues, Sharon de Toledo Martins, Lysangela Ronalte Alves, Igor C. Almeida, Leonardo Nimrichter

**Affiliations:** a Laboratório de Glicobiologia de Eucariotos, Departamento de Microbiologia Geral, Instituto de Microbiologia, Universidade Federal do Rio de Janeiro, Rio de Janeiro, Brazil; b Department of Biological Sciences, Border Biomedical Research Center, University of Texas at El Paso, El Paso, Texas, USA; c Laboratório de Ultraestrutura Celular Hertha Meyer, Instituto de Biofísica Carlos Chagas Filhos (IBCCF), Universidade Federal do Rio de Janeiro, Rio de Janeiro, Brazil; d LPO-COPEA, Instituto de Ciências Biomédicas & Centro Nacional de Biologia Estrutural e Bioimagem (CENABIO), Universidade Federal do Rio de Janeiro, Rio de Janeiro, Brazil; e Laboratório de Citotoxicidade e Genotoxicidade, Departamento de Bioquímica, Instituto de Química, Universidade Federal do Rio de Janeiro, Rio de Janeiro, Brazil; f Laboratório de Bioquímica e Imunologia das Micoses, Departamento de Microbiologia e Parasitologia, Instituto Biomédico, Universidade Federal Fluminense, Niterói, Brazil; g Department of Microbiology and Immunology, Stony Brook University, Stony Brook, New York, USA; h Department of Microbiology and Immunology and Division of Infectious Diseases, Stony Brook University, Stony Brook, New York, USA; i Veterans Affairs Medical Center, Northport, New York, USA; j Department of Microbiology and Immunology, Albert Einstein College of Medicine, Bronx, New York, USA; k Division of Infectious Diseases, Department of Medicine, Albert Einstein College of Medicine, Bronx, New York, USA; l Instituto Carlos Chagas (ICC), Fundação Oswaldo Cruz (FIOCRUZ), Curitiba, Brazil; m Centro de Desenvolvimento Tecnológico em Saúde (CDTS), Fundação Oswaldo Cruz, Rio de Janeiro, Brazil; n Instituto de Microbiologia Paulo de Góes, Universidade Federal do Rio de Janeiro, Rio de Janeiro, Brazil; University of Wisconsin—Madison; Leibniz Institute for Natural Product Research and Infection Biology–Hans Knoell Institute Jena (HKI)

**Keywords:** biofilm, *Candida albicans*, extracellular vesicles, lipids, yeast-to-hypha inhibition

## Abstract

In this study, we investigated the influence of fungal extracellular vesicles (EVs) during biofilm formation and morphogenesis in Candida albicans. Using crystal violet staining and scanning electron microscopy (SEM), we demonstrated that C. albicans EVs inhibited biofilm formation *in vitro*. By time-lapse microscopy and SEM, we showed that C. albicans EV treatment stopped filamentation and promoted pseudohyphae formation with multiple budding sites. The ability of C. albicans EVs to regulate dimorphism was further compared to EVs isolated from different C. albicans strains, Saccharomyces cerevisiae, and Histoplasma capsulatum. C. albicans EVs from distinct strains inhibited yeast-to-hyphae differentiation with morphological changes occurring in less than 4 h. EVs from S. cerevisiae and H. capsulatum modestly reduced morphogenesis, and the effect was evident after 24 h of incubation. The inhibitory activity of C. albicans EVs on phase transition was promoted by a combination of lipid compounds, which were identified by gas chromatography-tandem mass spectrometry analysis as sesquiterpenes, diterpenes, and fatty acids. Remarkably, C. albicans EVs were also able to reverse filamentation. Finally, C. albicans cells treated with C. albicans EVs for 24 h lost their capacity to penetrate agar and were avirulent when inoculated into Galleria mellonella. Our results indicate that fungal EVs can regulate yeast-to-hypha differentiation, thereby inhibiting biofilm formation and attenuating virulence.

## INTRODUCTION

Candida albicans is a common colonizer of the human skin and mucosa (1–3). However, in circumstances where the epithelial barrier, the immune system, and/or the microbiome are compromised, this species can overgrow and cause diseases that range from superficial to disseminated, life-threatening infections (4–6). The ability of C. albicans to colonize host tissues is associated with a combination of virulence factors and fitness attributes (4). Among them, a major strategic feature that facilitates C. albicans’s ability to persist in different environments is the capacity to switch morphological stages, which is a tightly regulated process called dimorphism, developed by fungal organisms (7).

Different environmental stimuli can induce dimorphism in C. albicans. For instance, yeast-to-hypha differentiation is stimulated by *N*-acetylglucosamine (GlcNAc), neutral pH, serum supplementation at 37°C, 5% CO_2_, low nitrogen and amino acids, and diverse other factors (8–12). Dimorphism in C. albicans can also be controlled by quorum sensing (QS), a mechanism of microbial communication (13, 14). Farnesol (FOH), farnesoic acid, and tyrosol are major morphogenesis-related QS molecules (QSMs) produced by C. albicans (15–17), although other alcohols derived from amino acids have been also characterized (18). FOH and farnesoic acid prevent yeast-to-hypha differentiation with no effects on growth rates (17–19). Tyrosol stimulates germ tube and hypha formation (15). These QSMs are also able to regulate biofilm formation (20, 21). The ability of free fatty acids to inhibit C. albicans filamentation and biofilm formation has also been reported (22–24).

Since the first description of fungal extracellular vesicles (EVs) by Rodrigues et al. in 2007 (25), studies have shown that fungal organisms use these compartments as a mechanism of molecular exportation (26–39). Heterogeneous populations of EVs containing lipids, proteins, polysaccharides, pigments, and nucleic acids cross the cell wall outward, reaching the extracellular environment (25, 28, 31, 33, 38, 40). Their implication during pathological and physiological processes is under investigation by a number of groups (29, 37, 41–43). Recently, Bielska and colleagues have shown that EVs isolated from a virulent strain of Cryptococcus gattii are efficiently taken up by macrophages previously infected with a nonvirulent strain of the same species (29). Inside phagocytes, the EVs derived from the virulent strain promoted rapid intracellular fungal growth and transferred virulence characteristics to the nonvirulent strain through a mechanism dependent on intact vesicles. The presence of stable RNA and proteins in the EVs was required (29). EVs also have a critical role during biofilm matrix production and drug resistance in C. albicans (44, 45).

In this work, we demonstrate that EVs produced by C. albicans cells (C. albicans EVs) impacted biofilm formation, yeast-to-hypha differentiation, and virulence. Our results reveal previously unknown roles of fungal EVs that impact the physiology and pathogenesis of C. albicans.

## RESULTS

### EVs produced by C. albicans inhibit biofilm formation.

**FIG 1:**
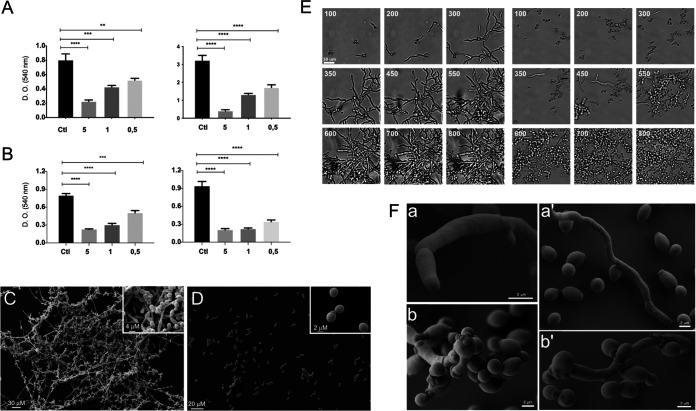
EVs from C. albicans inhibit biofilm formation and yeast-to-hyphae differentiation. Both protocols described in Materials and Methods for EV isolation were used with identical results. The results presented here were obtained from C. albicans 90028 EVs (yeast cells) isolated from liquid culture medium. (A) C. albicans (90028) (10^5^ yeasts) were inoculated into RPMI-MOPS in the presence or absence of C. albicans 90028 EVs. Different concentrations of EVs (0.5, 1, and 5 μg/mL, based on sterol content) were added to wells containing C. albicans (90028) yeasts, and cells were incubated to develop biofilm for 24 and 48 h in 96-well microplates. (B) C. albicans (90028) (10^5^ yeasts) were inoculated into RPMI-MOPS and incubated for 90 min. The nonadherent cells were washed out, and fresh medium containing C. albicans 90028 EVs (0.5, 1, and 5 μg/mL, based on sterol content) was added. PBS was used as control. Biofilm formation was quantified by crystal violet method. Group comparisons were submitted to one-way analysis of variance (ANOVA) with Dunnett’s correction (**, *P* < 0.002; ***, *P* < 0.001; ****, *P* < 0.0001). (C and D) For SEM, C. albicans (strain 90028) yeast cells, untreated (C) or treated with C. albicans EVs (5 μg/mL) (D), were inoculated onto coverslips previously coated with poly-l-lysine in RPMI-MOPS and incubated for 24 h. SEM images show the effect of EVs after 24 h of growth. Insets in panels C and D depict higher magnification of each condition. Bar, 30 μm. (E) C. albicans (90028) yeasts were inoculated into M199 medium to induce filamentation in the presence or absence of C. albicans 90028 EVs (5 μg/mL, based on sterol content). Cellular density was monitored, and the pictures represent the minutes of incubation (from 100 to 800 min). Bar, 30 μm. Growth under control conditions (absence of C. albicans 90028 EVs) and in the presence of C. albicans 90028 EVs. The numbers indicate the time in minutes. Bar, 30 μm. (F) C. albicans (90028) yeasts were inoculated into M199 medium to induce filamentation in the presence or absence of EVs (5 μg/mL, based on sterol content). SEM of cells after 24 h showing the hyphae and yeasts under control conditions (a and a′) in contrast with C. albicans 90028 EVs that led to the presence of pseudohyphae with multiple budding sites and yeasts (b and b′).

We began our studies using two distinct approaches to investigate the influence of fungal EVs during biofilm formation. Initially, C. albicans yeasts and EV derived from C. albicans strain 90028 were added to wells at the same time. Our results showed that the presence of yeast-derived C. albicans EVs negatively impacted biofilm formation in a dose-dependent manner (Fig. 1A). The highest density of EVs (sterol concentration corresponding to 5 μg/mL) reduced optical density (OD) measurements by approximately 80 to 85% compared to controls with no C. albicans EV stimulation. In a second test, to avoid any influence of EVs during the adhesion step, yeasts were added to the wells and incubated for 90 min. Then, the nonadherent cells were removed, and the EVs were added. Similar results were observed, suggesting that the activity of the EVs was independent of the adhesion step (Fig. 1B). SEM images obtained after 24 h of growth (experimental conditions of Fig. 1A) showed that the biofilm formed in the absence of C. albicans EVs (control condition) exhibited a standard architecture, with the presence of hyphae, pseudohyphae, and yeast cells attached to the coverslip (Fig. 1C). In contrast with control conditions where the three morphological stages were found, few yeast cells were visualized attached to the coverslip under the condition with EVs present (Fig. 1D). Thus, under the conditions used in our experiments, EVs significantly reduced yeast cell attachment and impaired biofilm formation in C. albicans. EV concentrations used in these experiments were initially based on previous studies performed by Bielska and colleagues (29).

### EVs from C. albicans inhibit yeast-to-hypha differentiation.

**FIG 2:**
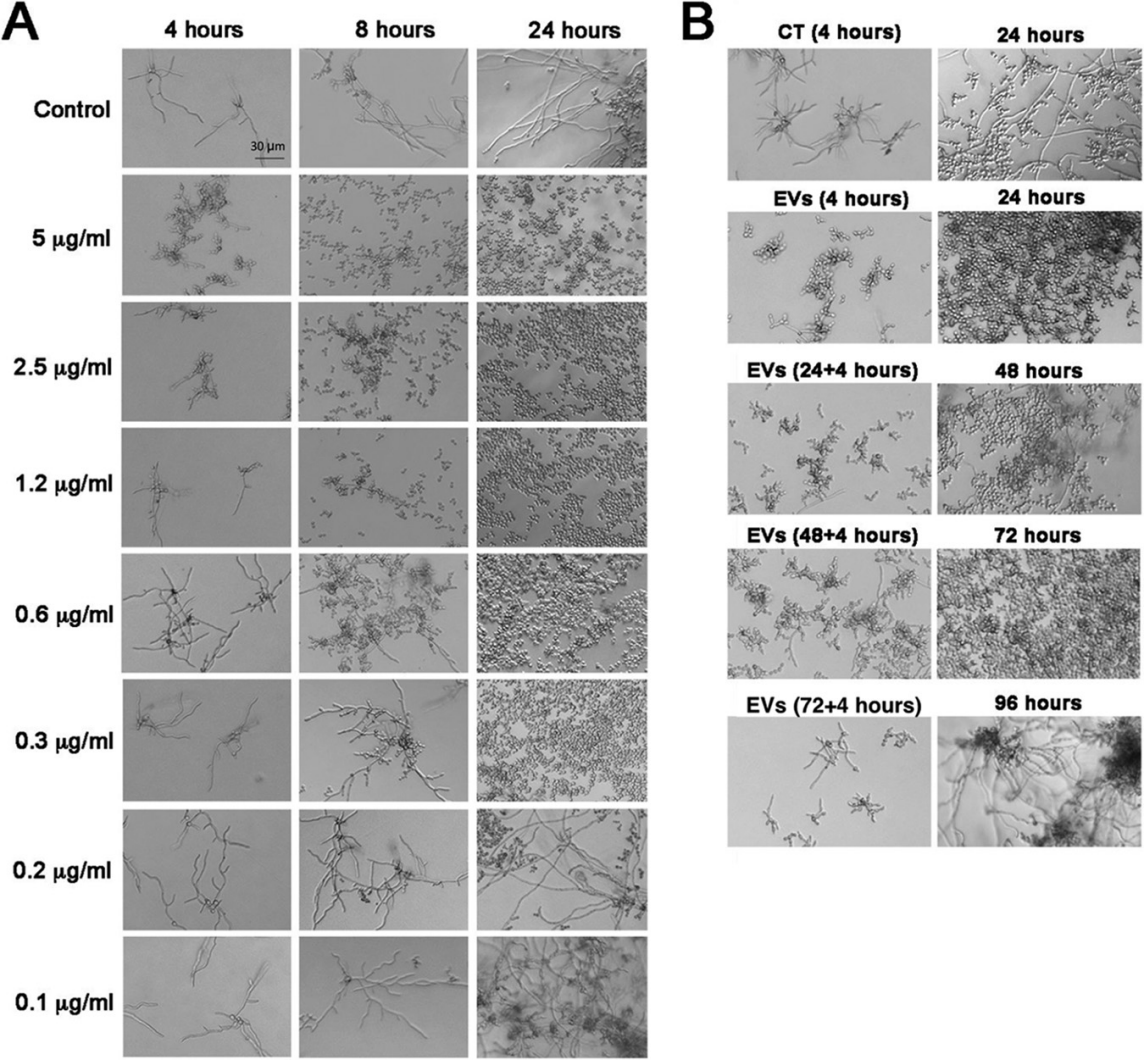
The inhibitory effect of EVs from C. albicans (strain 90028) on yeast-to-hypha conditions is dose dependent and has a long-term effect. (A) C. albicans (90028) yeasts were inoculated into M199 (pH 7) in the presence or absence of C. albicans 90028 EVs in different concentrations (equivalent to 0.1, 0.2, 0.3, 0.6, 1, 1.2, 2.5, and 5 μg sterol/mL). Bar, 30 μm. (B) The inhibitory effect of a single C. albicans EV treatment was tested after 24, 48, 72, and 96 h. Briefly, overnight-treated yeasts were washed with sterile PBS, and aliquots of (2.5 × 10³ cells/well) cells were transferred to fresh M199 medium. Yeasts were incubated under the same conditions without addition of EVs for 24 h, and the differentiation was accompanied microscopically. This step was repeated until the reestablishment of C. albicans filamentation. Bar, 30 μm. Experiments were performed in biological triplicate with consistent results.

The ability of C. albicans 90028 EVs to inhibit biofilm formation suggested that EVs could control morphogenesis in C. albicans strain 90028. We then monitored microscopically the impact of C. albicans EVs on yeast-to-hypha differentiation induced by M199 medium at 37°C. In the absence of C. albicans EVs (control condition), the yeast cells adhered to the solid substrate (Fig. 1E, left) and formed germ tubes after 1 to 2 h of incubation. This step was followed by true hypha formation. Few branching hyphae and yeast cells were visualized after 8 h, but hyphae represented the predominant morphological stage. The time-lapse microscopy of 18 h of incubation is presented as Movie S1 at 10.6084/m9.figshare.19403894. In contrast, the addition of EVs drastically changed the phenotype observed (Fig. 1E, right, and Movie S2 at 10.6084/m9.figshare.19403894). Although the yeast cells were attached to the plate and started to form germ tubes, the development of true hypha after elongation was not observed (Fig. 1E, right, and Movie S2 at 10.6084/m9.figshare.19403894). Instead, the appearance of constrictions on elongated cells suggested the development of pseudohyphae after 4 to 7 h. In addition, the cells exposed to C. albicans 90028 EVs lost their ability to adhere to the well after 6 h of incubation (Movie S2). Multiple budding points, potentially related to faster growth, were observed along the pseudohyphae, and after 24 h, only yeast cells were detected (Fig. 1E, right, and Fig. 1F). Long pseudohyphae filaments with yeast cells clusters were observed, including a large number of yeast cells derived from pseudohyphae (Fig. 1E, right). Furthermore, there was no indication of cytotoxicity under these conditions since all the yeast cells were able to grow (Movie S2 at 10.6084/m9.figshare.19403894). SEM images show the ultrastructural details of pseudohyphae. Multiple budding yeasts and several bud scars were observed in the experiments with C. albicans 90028 EVs added (Fig. 1F). This phenomenon of yeast induction occurred in a dose-dependent manner. A minimum density of C. albicans EVs, equivalent to 0.3 to 0.6 μg sterol/mL, was required to inhibit morphogenesis at least partially (Fig. 2A). However, the inhibitory activity was not dependent on the number of yeast cells since different cell suspensions (1.5 × 10^3^ to 3.5 × 10^4^ yeast cells/well) were similarly affected using the same density (5 μg sterol/mL) of EVs (data not shown).

Remarkably, a single treatment with EVs was sufficient to impact the yeast-to-hypha differentiation for at least 72 h (Fig. 2B). Aliquots of pretreated yeast cells (2.5 × 10^3^ yeast cells/well) were washed in phosphate-buffered saline (PBS) and plated onto fresh and EV-free M199 medium. The culture was monitored microscopically for 24 h and then transferred again to fresh medium without additional EVs. This process was repeated three times, and aliquots from 48 and 72 h were added to fresh M199 medium for continued microscopic monitoring. Regular hypha formation was observed only after 96 h, 4 days after the initial addition of EVs.

To certify that the inhibitory effect of C. albicans 90028 EVs was not due to coisolated contaminants, we fractioned our bioactive samples using size exclusion chromatography (SEC) in a Sepharose CL-4B column. A single peak (fractions 8 to 10) containing proteins and lipids was observed (Fig. S1A and B at 10.6084/m9.figshare.19403894). Dynamic light scattering (DLS) analysis confirmed the presence of EVs in fraction 8 (Fig. S1C at 10.6084/m9.figshare.19403894), corresponding to the most concentrated sample with larger amounts of lipids and proteins, but not in the adjacent ones. Two populations were characterized with average sizes previously reported in our studies (31), ranging from 50 to 120 and 240 to 350 nm, and possibly corresponding to exosomes and ectosomes/microparticles, respectively. The inhibitory activity of the lipids from each fraction was then evaluated. The addition of fraction 8 to yeast cultures inhibited the yeast-to-hypha differentiation of C. albicans after 6 h of incubation (Fig. S1D at 10.6084/m9.figshare.19403894). In contrast, fraction 10, where the protein and lipid concentrations were significantly reduced compared to fraction 8, did not modify morphogenesis as efficient hyphae formation occurred. The other fractions in which lipids and proteins were not detected did not inhibit phase transitions (data not shown).

### General properties of EVs produced by different C. albicans strains.

**FIG 3:**
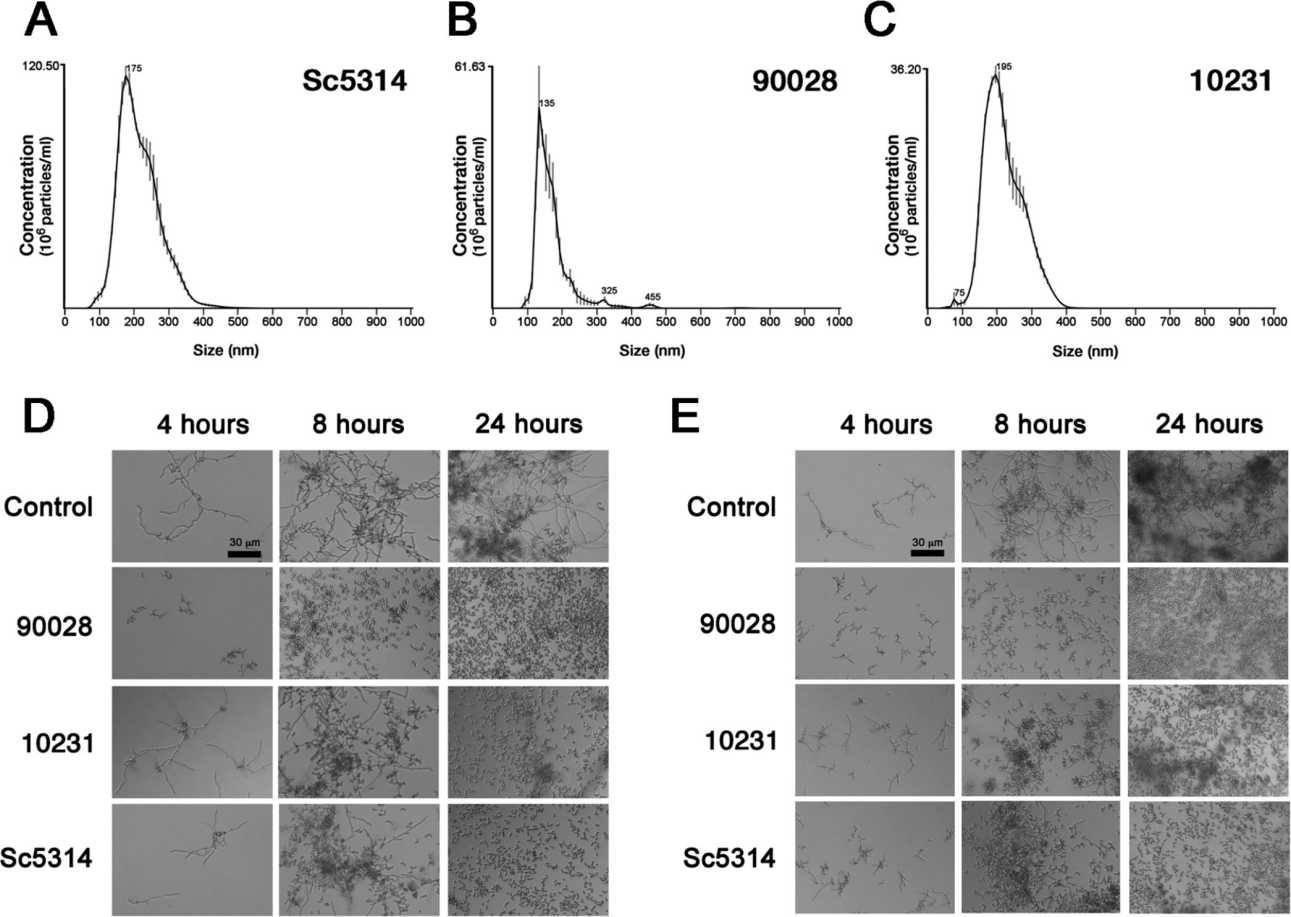
EVs from different C. albicans strains inhibit yeast-to-hyphae transition of strains 90028 and 10231. NTA profile of EVs produced by C. albicans strains SC5314 (A), 90028 (B), and 10231 (C) showed similar properties of EVs. C. albicans yeast cells of strains 90028 (D) or 10231 (E) were inoculated into M199 (pH 7) in the presence or absence of C. albicans EVs (equivalent to 5 μg sterol/mL) from distinct strains of C. albicans (90028, 10231, and SC5314). The effects were visualized after 4, 8, and 24 h. PBS was added alone as a control. Bar, 30 μm. Experiments were performed four times with consistent results.

EVs produced by different strains of C. albicans were isolated from culture supernatants and compared using nanoparticle tracking analysis (NTA) (46). The size distribution of the EVs produced by C. albicans strains used in this work ranged from 100 to 500 nm (Fig. 3A to C), similar to measurements published for EVs released by C. albicans and other fungal species (31, 46). EVs released by strains SC5314 and 10231 were comparable to each other, ranging from 100 to 400 nm (Fig. 3A and C). EVs produced by strain 90028 were mainly composed of smaller particles, ranging between 100 to 250 nm, but also contained minor populations between 300 and 350 nm and 420 to 480 nm (Fig. 3B). Considering that the C. albicans strains released EVs with distinct average size, we normalized our experiments using the total content of sterol in each vesicle preparation, as used in previous studies from our group (31, 34). Of note, this method is highly reproducible and requires small amounts of samples.

### The ability of EVs to inhibit yeast-to-hypha differentiation is reproducible in other strains of C. albicans.

We investigated whether the ability to inhibit yeast-to-hypha differentiation was reproducible in EVs obtained from other strains of C. albicans. Thus, EVs were isolated from strains with distinct abilities to cause infection. For instance, strain 10231 is less virulent and does not produce FOH (47). Instead, it produces farnesoic acid, an FOH derivative at least 10-fold less active in its ability to inhibit yeast-to-hypha conversion (19). Strains 90028 and SC5314, on the other hand, are regular producers of FOH (17). The results presented in Fig. 3D showed that C. albicans EVs from 10231 and SC5314 inhibited hypha formation of strain 90028. However, EVs from these other strains exhibited a reduced effect at the initial times of incubation compared to C. albicans 90028 EVs, indicating that although each strain’s EVs had the ability to inhibit filamentation, they may have a distinct potency. To confirm that the effect was not strain specific, we evaluated the effect of the EVs produced by strains 10231 and 90028 on the morphogenesis of strain 10231 (Fig. 3E). The EV-mediated inhibition of filamentation of strain 10231 was confirmed. The recovery of EVs was similar using solid or liquid medium (average of 0.16 and 0.18 μg of sterol/10^9^ cells, respectively). Further experiments were performed using EVs produced by the 90028 strain cultivated in liquid medium since this condition resulted in better yields of EVs.

### EV molecules with yeast-to-hypha inhibitory activity are thermoresistant and enriched in preparations containing nonpolar lipids.

**FIG 4:**
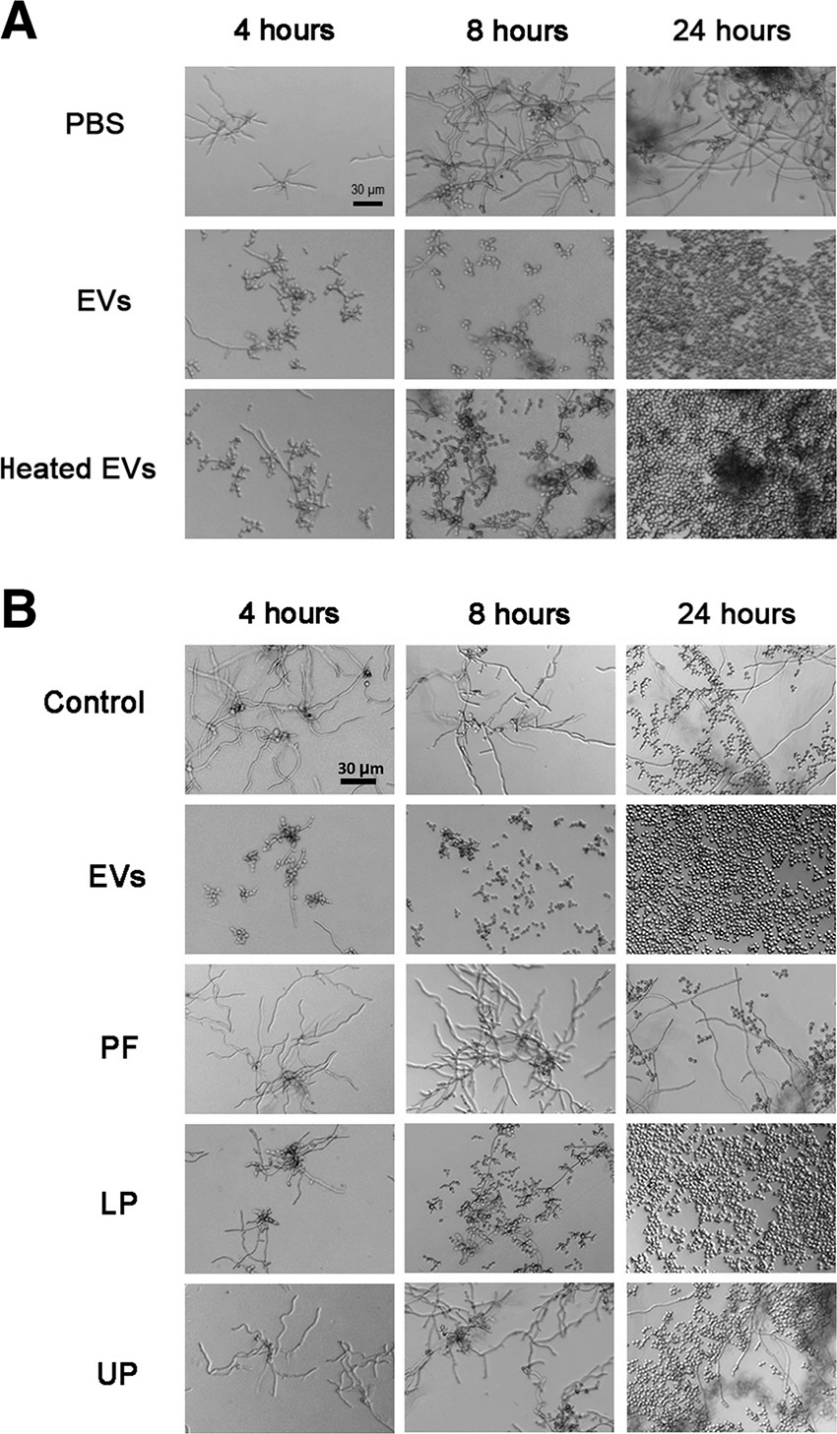
Thermoresistant molecules carried by C. albicans EVs are associated with yeast-to-hyphae inhibitory effect. (A) C. albicans (90028) yeast cells were inoculated into M199 (pH 7) in the presence or absence of C. albicans 90028 EVs or heat-treated EVs in a concentration equivalent to 5 μg sterol/mL. The effect was visualized after 4, 8, and 24 h. PBS was added alone as a control. Bar, 30 μm. The results shown are representative of three independent experiments. (B) C. albicans (90028) yeasts were inoculated into M199 in the presence or absence of C. albicans EVs, EV-derived protein-rich fraction (PF), or lipid lower (LP) or upper phase (UP) in a concentration equivalent to 5 μg sterol/mL. The effect was visualized after 4, 8, and 24 h. PBS was added alone as a control. Bar, 30 μm.

The ability to prevent yeast-to-hypha differentiation was preserved by C. albicans 90028 EVs after heating them at 90°C for 15 min (Fig. 4A), suggesting that thermoresistant molecules could be mediating the inhibitory effect. Since lipids participate in C. albicans morphogenesis (16, 21), we extracted and fractioned the lipid components contained in C. albicans 90028 EVs and tested their inhibitory activity. The crude lipid extract was partitioned using standardized protocols to enrich lipids according to their polarity (48), and the organic (lower and upper) phases were added to yeast suspensions in M199 medium for microscopic monitoring of morphogenesis. The lower phase (LP) activity was very similar to C. albicans 90028 EVs in its ability to inhibit differentiation (Fig. 4B). Although the upper phase (UP) was also active, its effect was observed only after 24 h of incubation. The precipitate (protein-rich fraction [PF]) obtained from C. albicans 90028 EV lipid extraction, likely rich in proteins, had no apparent influence on morphogenesis (Fig. 4B). The LP of lipids extracted from the whole cells were also able to inhibit C. albicans differentiation (data not shown). LP obtained from liquid Sabouraud (no yeast cells, control medium) using the same extraction and partition protocol showed no activity (data not shown).

### EVs produced by other fungal species affect yeast-to-hypha differentiation in C. albicans.

Based on the lipid nature of the bioactive molecules studied in our work and the fact that FOH and farnesoic acid are QSM lipids that control yeast-to-hypha differentiation in C. albicans (16, 21, 23), we hypothesized that the presence of FOH and farnesoic acid in EVs from C. albicans could result in filamentation inhibition. To confirm this possibility, we asked whether EVs from S. cerevisiae, which synthesizes and exports FOH in very small amounts (49), would regulate morphogenesis in C. albicans. We also included EVs from H. capsulatum in these analyses since FOH or FOH derivatives have not been characterized as lipid components in this fungus. We first investigated whether the strains used in our study produced detectable amounts of FOH into the LP by reverse-phase thin-layer chromatography (TLC). Whole cells were used in order to obtain larger amounts of lipids. The TLC showed a sharp band in the LP of C. albicans (strain 90028) with identical color and retention factor (*R_f_*) obtained for an FOH standard, strongly indicating the presence of this molecule in the C. albicans extract (Fig. S2A at 10.6084/m9.figshare.19403894). A band with a similar *R_f_* was observed in the LP of H. capsulatum; however, a distinct yellowish color was visualized (Fig. S2A). Thus, a more detailed structural analysis was necessary to confirm the chemical nature of that band, as further described in this section. No molecules migrating as FOH were detected in the LP from S. cerevisiae. Nevertheless, EVs from these three species were able to impair C. albicans hypha formation (Fig. S2B at 10.6084/m9.figshare.19403894). Clearly, the kinetics of inhibition (comparing 8 h versus 24 h of incubation time) observed for EVs released by H. capsulatum and S. cerevisiae, as well as for C. albicans 10231 EVs, were considerably slower than C. albicans 90028 EVs. With H. capsulatum EVs, the inhibitory effect was observed only after an overnight incubation. The first signals of inhibitory effect from S. cerevisiae EVs were detected after 8 h of incubation, an effect comparable with EVs from C. albicans strain 10231 (Fig. 3D). To confirm that the inhibitory effect was not pleiotropic, caused by EVs produced by any of the organisms studied, we also tested the activity of EVs from murine macrophages-like (MOs). Contrasting with the activity of fungal EVs, MO EVs did not interfere with yeast-to-hypha differentiation *in vitro* (Fig. S2B at 10.6084/m9.figshare.19403894). The fact that EVs lacking or expressing reduced levels of FOH inhibit C. albicans differentiation, even at a lower efficacy, suggests that other EV molecules, in addition to FOH, also participate in the inhibitory mechanisms. We next investigated whether other lipids could be potentially involved in the inhibitory effect of EVs.

### Terpenes and fatty acids are the major compounds potentially involved with C. albicans yeast-to-hypha inhibition.

To investigate whether other lipids produced by C. albicans 90028 could mediate the inhibitory activity of C. albicans EVs, we tested the distinct TLC bands from the C. albicans LP extract. Using a preparative TLC plate and staining the lipids with mild iodine vapor, to avoid lipid degradation, we obtained five major fractions (A to E) corresponding to separate bands (Fig. S3 at 10.6084/m9.figshare.19403894). The ability of each fraction to inhibit yeast-to-hypha differentiation was investigated, and only one (band A), with an *R_f_* similar to an authentic FOH standard, showed an active effect (Fig. S3).

**FIG 5:**
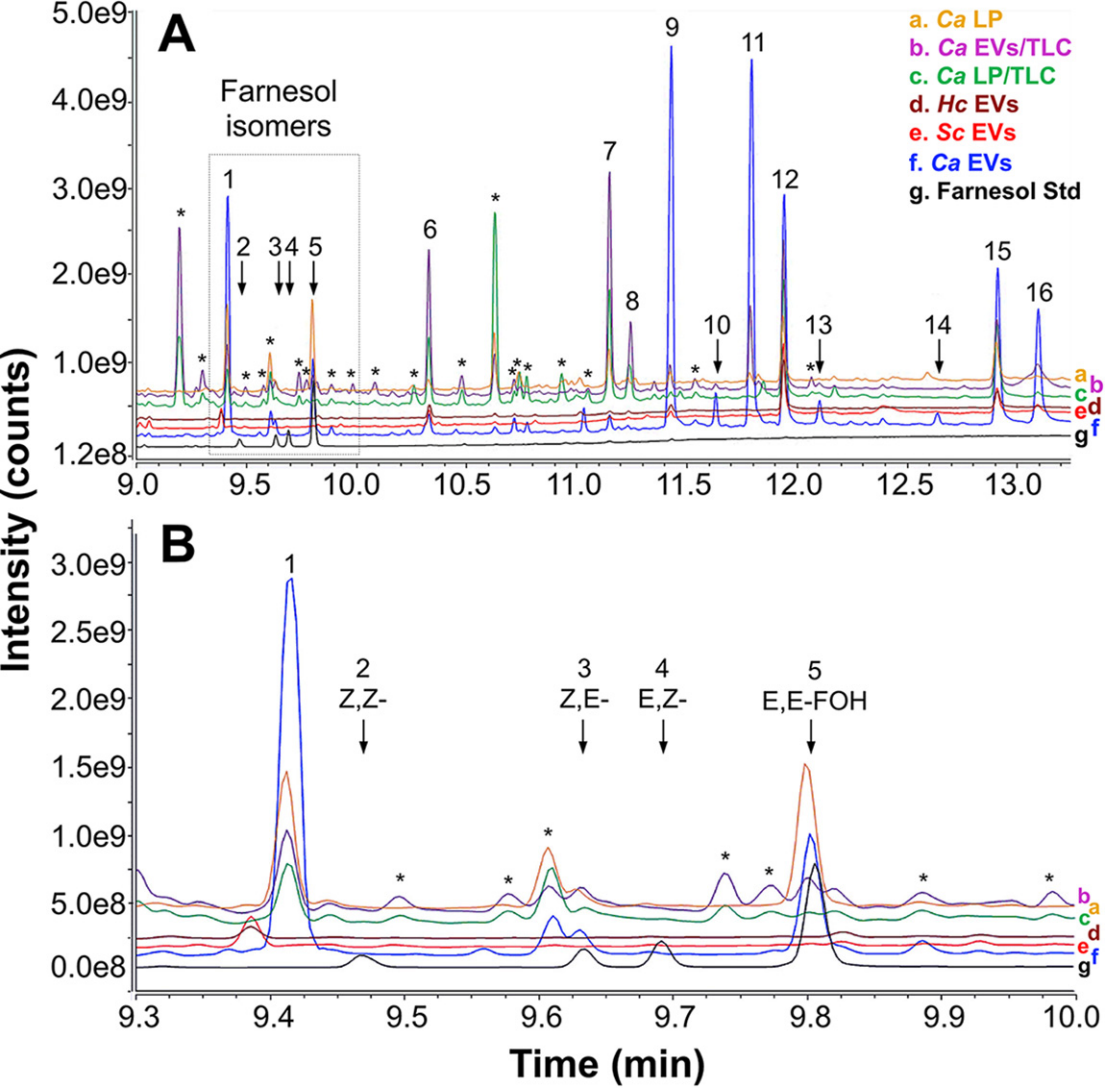
GC-MS analysis of fungal lipids and EVs. Lipids were extracted from the C. albicans 90028 LP fraction and EVs and from the RP-TLC band comigrating with farnesol in the two samples. Lipids were also extracted from H. capsulatum and S. cerevisiae EVs. The resulting samples were analyzed by GC-MS/MS. (A) GC-MS full-scan chromatogram. (B) The chromatogram region containing the four farnesol isomers is indicated (dashed rectangle) and shown in detail. A standard mixture containing the four farnesol isomers (Z,Z-, Z,E-, E,Z-, and E,E-FOH) was used as reference (black line/trace). The GC-MS full-scan traces were overlaid and labeled (a to g) to facilitate visualization and comparison. Farnesol isomers and peaks of interest are indicated numerically and annotated in Table 1. Asterisks indicate contaminants (e.g., phthalates and other plasticizers) or compounds not identified in the library (low structural match probability). Ca, C. albicans; Hc, H. capsulatum; Sc, S. cerevisiae.

We then concentrated our efforts on the analysis of FOH in fungal EVs and lipid extracts. To that end, we followed a protocol previously used for FOH extraction and employed a highly sensitive gas chromatography-mass spectrometry with selected reaction monitoring (GC-MS/SRM) method, using an external bona fide standard of FOH (50, 51). As observed in Fig. 5A and B (black line), the FOH standard (at 4.5 pmol/μL, 1 μL injection) is comprised of a mixture of four isomers (2-*cis*,6-*cis*-farnesol [Z,Z-FOH], 2-*cis*,6-*trans*-farnesol [Z,E-FOH], 2-*trans*,6-*cis*-farnesol [E,Z-FOH], and 2-*trans*,6-*trans*-farnesol [E,E-FOH]), which clearly separated from each other using the GC gradient conditions employed. The isomer profile was comparable to that previously reported (51). Under the experimental conditions used for the standard mixture, we detected peaks (at 9.65 and 9.81 min) with SRM transitions (not shown) and full-scan chromatogram elution times similar to Z,E-FOH and E,E-FOH isomers (peaks 3 and 5, respectively) in the C. albicans 90028 yeast EV sample (Fig. 5A and B). Table 1 shows the relative peak intensity of these FOH isomers and other identified compounds found in fungal EVs and other lipid fractions. Besides the two FOH isomers, the GC-MS analysis revealed that C. albicans 90028 EVs contained large amounts of other terpenoids or isoprenoids such as geranylgeraniol isomers and, to a much lesser extent, squalene (Table 1).

**TABLE 1:** Major lipid compounds identified by GC-MS/MS in the lipid LP fractions and EVs of C. albicans and EVs of H. capsulatum and S. cerevisiae

Peak no.	RT (min)	Compound	Peak relative intensity (%) of fungal preparation/fraction:					
			C. albicans				H. capsulatum	S. cerevisiae
			LP	EVs	LP/TLC	EVs/TLC	EVs	EVs
1	9.42	2,3-Dihydro-6-*trans*-farnesol (6,10-dodecadien-1-ol,3,7,11 trimethyl)	93	63	32	25	ND^*a*^	ND
2	9.47	2,6-*Cis*-farnesol (Z,Z-farnesol)	ND	ND	ND	ND	ND	ND
3	9.63	2-*Cis*,6-*trans*-farnesol (Z,E-farnesol)	ND	4	ND	7	ND	ND
4	9.68	2-*Trans*,6-*cis*-farnesol (E,Z-farnesol)	ND	ND	ND	ND	ND	ND
5	9.81	2,6-*Trans*-farnesol (E,E-farnesol)	100	20	5	9	ND	ND
6	10.33	Dodecanoic acid (lauric acid, C_12:0_)	14	5	57	67	25	23
7	11.15	Tetradecanoic acid (myristic acid, C_14:0_)	41	4	97	100	6	4
8	11.25	Dodecanoic acid, undecyl ester (C_23:0_-O_2_)	ND	ND	30	32	6	4
9	11.42	2,3-Dihydro-6-*trans*-geraniol (6,10,14-hexadecatrien-1-ol 3,7,11,15 tetramethyl)	14	100	8	12	19	9
10	11.63	Geranylgeraniol (isomer 1)^*b*^	ND	9	ND	3	ND	ND
11	11.79	Geranylgeraniol (isomer 2)^*b*^	10	97	ND	39	ND	ND
12	11.93	Ascorbic acid 2,6-dihexadecanoate	72	60	100	68	100	100
13	12.11	(9Z)-hexadec-9-enoic acid (palmitoleic acid, C_16:2_)	ND	7	3	3	ND	4
14	12.64	Squalene	ND	3	ND	ND	ND	ND
15	12.92	Octadecanoic acid (stearic acid, C_18:0_)	41	41	62	30	38	36
16	13.12	Octadecenoic acid (oleic acid, C_18:1_)	3	30	5	10	ND	14

a ND, not detected (below the method's limit of detection).

b We were unable to determine the precise structures of these geranylgeraniol geometric isomers through fragmentation spectral library matching.

Using the standard calibration curve from the d6-E,E-FOH isomer, we quantified the amounts of each isoprenoid/terpenoid found in the fungal EVs (Table 2), confirming the results above and showing a significant enrichment of isoprenoids in EVs carried by C. albicans strain 90028. The FOH derivative 2,3-dihydro-6-*trans*-farnesol (6, 10-dodecadienol-1-ol, 3,7,11 trimethyl) (peak 1) and related isoprenoid, 2,3-dihydro-6-*trans*-geraniol (6,10,14-hexadecatrien-1-ol, 3,7,11,15 tetramethyl) (peak 9), corresponding to FOH and geranylgeraniol units lacking a double bond at C-2, respectively, were also characterized as major peaks (Fig. 5; Tables 1 and 2). The lipid content of C. albicans 10231 LP and EVs was also analyzed (Fig. S4 at 10.6084/m9.figshare.19403894). In the experimental conditions used, we could not detect any FOH or DHFOH isomers in the C. albicans 10231 LP and EVs, suggesting that the ability to produce FOH in this strain is completely abrogated (Fig. S4; Fig. 5; Tables 1 and 2). The peak observed at 9.58 min by GC-MS analysis, with similar retention time as Z,E-FOH, was identified as a contaminant in the NIST mass spectral library (Fig. S4 and Table S1 at 10.6084/m9.figshare.19403894). Of those isoprenoids, only 2,3-dihydro-6-*trans*-geraniol was also found in H. capsulatum and S. cerevisiae EVs. None of the FOH isomers or its derivative (2,3-dihydro-6-*trans*-farnesol [dihydrofarnesol or DHFOH]) were detected in the EVs from H. capsulatum and S. cerevisiae, confirming that these molecules are only enriched in C. albicans 90028 EVs (Fig. 5; Tables 1 and 2). Together, these data confirm that although the terpenes considerably increased the inhibitory activity of C. albicans 90028 EVs, they are not the only lipids able to control C. albicans filamentation.

**TABLE 2:** Quantification of FOH and FOH-like structures in lipid LP fractions and EVs of C. albicans and EVs from H. capsulatum and S. cerevisiae

	Farnesol isomers (ng/100 μg total lipid or protein) in compound:^*a*^			Farnesol-like structures (ng/100 μg total lipid or protein) in compound:^*a*^		
Fungal fraction/prepn	2,3-Dihydro-6-*trans-*farnesol	Z,E-FOH	E,E-FOH	2,3-Dihydro-6-*trans*-geraniol	Geranylgeraniol (isomer 1)	Geranylgeraniol (isomer 2)
C. albicans LP	16.3	1.8	19	2.1	ND	1.2
C. albicans LP/TLC	2.1	ND^*b*^	ND	ND	ND	ND
C. albicans EVs	33.7	1.6	9.7	67	4.1	63.4
C. albicans EVs/TLC	48.8	7.5	9.6	21	ND	77.4
H. capsulatum EVs	ND	ND	ND	4.7	ND	1.9
S. cerevisiae EVs	ND	ND	ND	1.2	ND	ND

a LP and LP/TLC fractions were normalized by 100 μg of total lipid, whereas all EV preparations were normalized by 100 μg of protein.

b ND, not detected (below the method's limit of detection).

The other class of lipids recognized to inhibit hyphal growth and biofilm formation in C. albicans is the fatty acids (22, 23, 52). In our analysis, five fatty acids were identified as major C. albicans 90028 EV components, including octadecanoic (stearic acid, C_18:0_) and octadecenoic (oleic acid, C_18:1_) acids, and, to a much lesser extent, (9Z)-hexadec-9-enoic (palmitoleic acid, C_16:1_), dodecanoic (lauric acid, C_12:0_), and tetradecanoic (myristic acid, C_14:0_) acids (Fig. 5 and Table 1). Remarkably, larger amounts of myristic and stearic acid were found enriched in C. albicans 10231 EVs, along with small amounts of pentadecyclic acid, C_16:1_ and C_18:1_ (Fig. S4). Fatty acids were also found in EVs from H. capsulatum (C_12:0_, C_14:0_, and C_18:0_) and S. cerevisiae (C_12:0_, C_14:0_, C_16:1_, C_18:0_, and C_18:1_) (Fig. 5 and Table 1). Large amounts of ascorbic acid 2,6-dihexadecanoate were found in EVs from all species except for the C. albicans strain 10231 (Fig. 5, Fig. S4, Table 1, and Table S1).

We also characterized the major lipids found in the LP from C. albicans and its bioactive TLC-derived band (Fig. S3). Equivalent amounts of DHFOH and E,E-FOH were detected in C. albicans 90028 LP (Table 1). The derivative 2,3-dihydro-6-*trans*-geraniol was also identified in C. albicans 90028 LP (Tables 1 and 2). Moreover, the free fatty acids C_12:0_, C_14:0_, C_18:0_, and C_18:1_ were also characterized in this fraction. Finally, we resolved the lipids from C. albicans 90028 EVs by RP-TLC, and the band corresponding to the FOH *R_f_* was also analyzed. We found a high similarity between the total EV lipids and this band, with a clear enrichment of FOH isomers and FOH-like structures and other isoprenoids/terpenoids (Tables 1 and 2). In addition, we observed an enrichment of the fatty acids C_12:0_ and C_14:0_, and dodecanoic acid undecyl ester (C_23:0_-O_2_) (Fig. 5A and Table 1).

### DHFOH and FOH similarly reproduce the effects of C. albicans EVs on filamentation.

**FIG 6:**
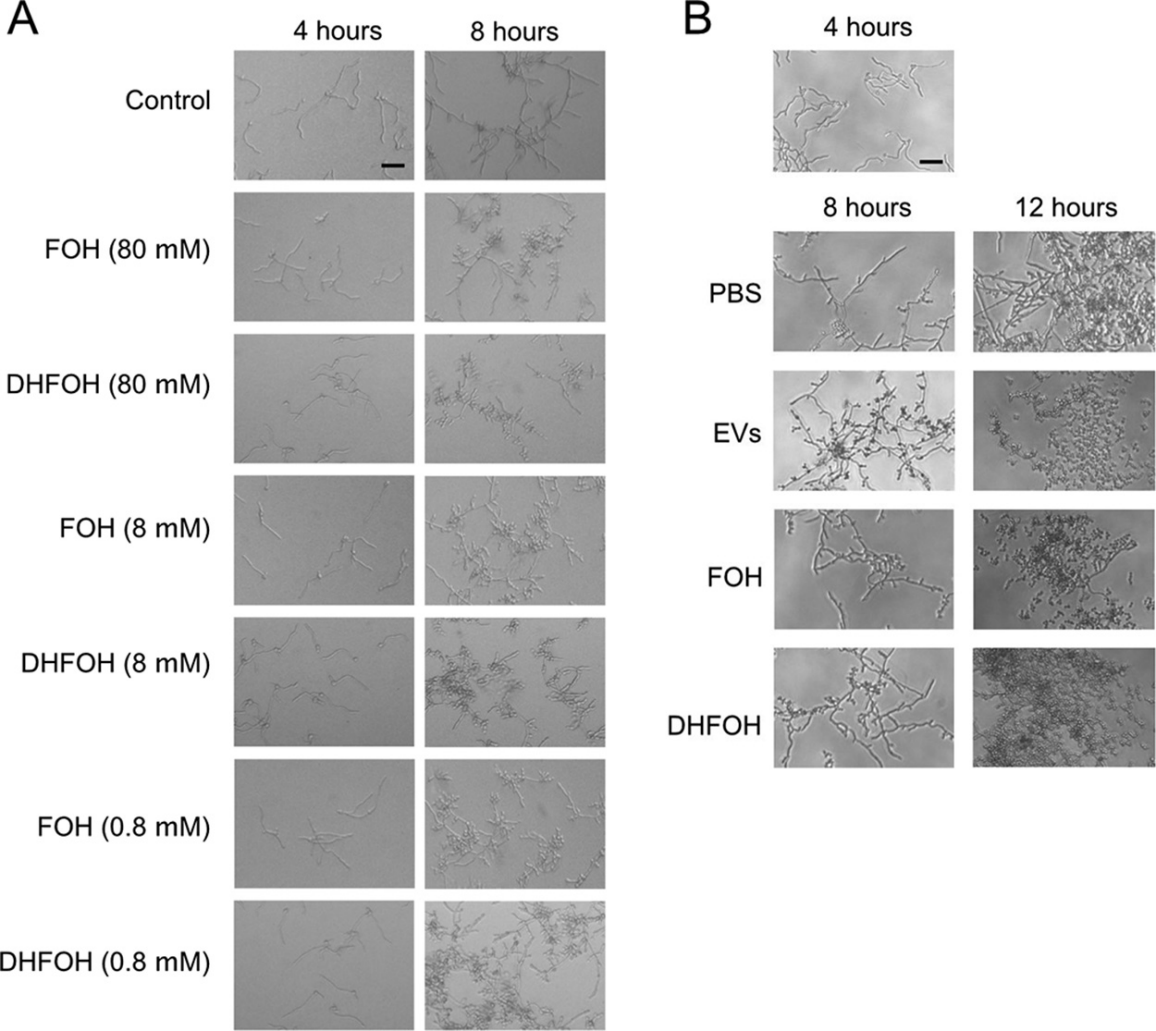
C. albicans EVs, FOH, and DHFOH reversed the yeast-to-hypha differentiation. (A) C. albicans (90028) yeast cells were inoculated into M199 (pH 7) in the presence or absence of distinct concentrations of FOH or DHFOH. (B) C. albicans (90028) yeasts were inoculated into M199 and incubated for 4 h to induce hyphae formation. Then, PBS (control), C. albicans EVs (5 μg/mL), FOH (25 μM), or DHFOH (25 μM) were added to the wells. Cell morphology was visualized after 8 and 12 h. Bars, 30 μm.

As mentioned previously, FOH is a major QSM produced by C. albicans with the ability to block filamentation (17, 53). Given that the major terpenes carried by EVs from C. albicans strain 90028 are DHFOH and 2,3-dihydro-6-*trans*-geraniol (Table 1), we investigated the efficacy of a commercially available, synthetic DHFOH to inhibit C. albicans filamentation. First, this standard was analyzed by GC-MS to confirm its purity and integrity (data not shown). The bona fide standard exhibited an activity similar to FOH (Fig. 6A and B). Of note, the biological activities of commercial FOH and DHFOH were considerably reduced within a few weeks in stock solution at −20°C (data not shown). On the other hand, synthetic geranylgeraniol and ascorbic acid 2,6-dihexadecanoate were not able to interfere with yeast-to-hypha differentiation (data not shown). Unfortunately, 2,3-dihydro-6-*trans*-geraniol, observed in C. albicans 90028 LP and EVs (Fig. 5 and Table 1), was not commercially available as synthetic or purified compound, and therefore, its biological activity could not be confirmed.

### EVs from C. albicans yeast cells reverse the yeast-to-hypha differentiation.

We next investigated whether C. albicans EVs were able to reverse the yeast-to-hypha differentiation induced by M199 medium. Yeast cells were plated onto M199, and hypha formation was observed after 4 h of induction (Fig. 6B), at which time C. albicans EVs, FOH, or DHFOH were added to the wells. Yeast cells were visualized 4 h after the addition of C. albicans EVs to the filamentous cultures, indicating that fungal EVs and the terpenes were able to efficiently reverse the yeast-to-hypha morphogenesis, even after the highly regulated process had been initiated.

### Inhibitory activity caused by C. albicans EVs is reversed by db-cAMP addition.

**FIG 7:**
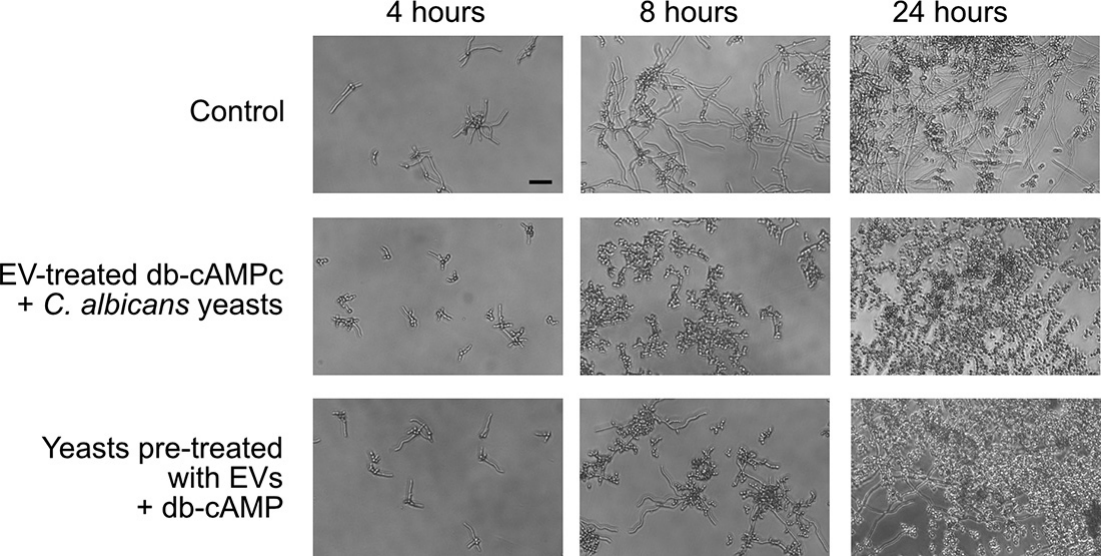
The reversing effect on C. albicans morphogenesis caused by db-cAMP is inactivated by preincubation with C. albicans EVs. db-cAMP preincubated with EVs (5 μg/mL) does not reverse the inhibitory effect of C. albicans 90028 EVs on C. albicans phase transition. The addition of db-cAMPc (10 mM) reverses the yeast-to-hyphae inhibition caused by C. albicans 90028 pretreated with C. albicans 90028 EVs. Bar, 30 μm.

FOH stops C. albicans filamentation by inhibiting adenylate cyclase (Cyr1) (54). This effect is rescued *in vitro* by the addition of exogenous db-cAMP (55). To investigate whether the EVs’ inhibitory effect is mediated by Cyr1 repression, we performed two experiments. First, we incubated db-cAMP and C. albicans 90028 EVs for 1 h and then added the mixture to C. albicans yeasts. Under these experimental conditions, the db-cAMP had no effect, and the inhibitory activity was similar to C. albicans EVs alone (Fig. 7). These results indicated that the EVs are not inhibiting Cyr1 and suggested that other signaling mechanisms could be involved. However, we cannot rule out that the EVs sequestered or hydrolyzed the db-cAMP. To address these possibilities, we first treated C. albicans yeasts with EVs for 1 h, and then db-cAMP was added to the system. Under these conditions, the filamentation was observed after 8 h, confirming that Cyr1 was targeted by C. albicans EV activity (Fig. 7). However, after 24 h, we observed that the control conditions (PBS) exhibited a higher proportion of hyphae than the conditions where db-cAMP was added to yeasts pretreated with EVs. These results suggested that although Cyr1 seems to be a major target for C. albicans EVs, other mechanisms might also be involved.

### Treatment with EVs reduces the ability of C. albicans to penetrate solid medium and decreases fungal virulence in a Galleria mellonella model of candidiasis.

**FIG 8:**
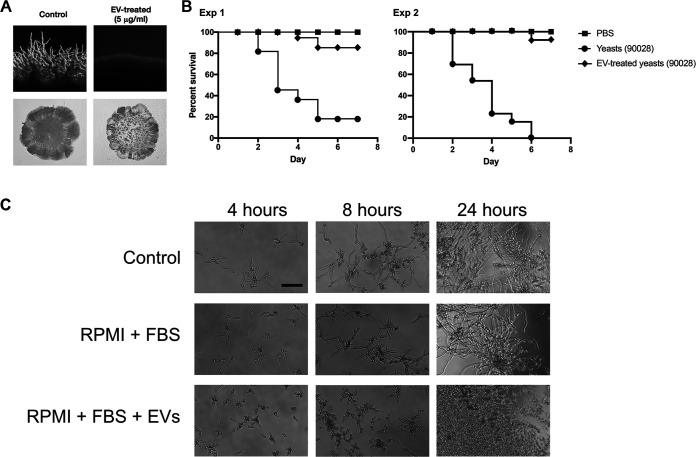
C. albicans EVs decrease the capacity of C. albicans to penetrate agar and attenuate virulence in Galleria mellonella. (A) C. albicans (90028) yeasts were treated with PBS (control) or C. albicans 90028 EVs in PBS (5 μg/mL) and then plated onto M199 agar for 7 days. The presence of filamentation (top panels) and agar invasion (bottom panels) was observed only under control conditions. (B) G. mellonella larvae were infected with 10^5^ C. albicans yeast untreated or pretreated with C. albicans 90028 EVs (5 μg/mL for 24 h), and survival was monitored (experiments 1 and 2). PBS indicates uninfected larvae injected with PBS. (C) C. albicans (90028) yeasts were inoculated into RPMI supplemented with FBS (10%) in the presence or absence of C. albicans EVs in a concentration equivalent to 5 μg sterol/mL.

Due to the well-established connections between pathogenesis and morphological transitions in C. albicans (12), we investigated whether treatment with C. albicans EVs could abrogate the ability of the fungus to penetrate solid medium, a consequence of filamentous growth regularly correlated with virulence (56). Colonies of C. albicans cultivated in M199 agar medium produced normal hypha at the edges after 5 to 6 days of invasive growth (Fig. 8A, top left panel). In contrast, after 5 to 6 days, no hyphae were visualized when yeast cells were pretreated with C. albicans EVs (Fig. 8A, top right panel). In fact, under C. albicans EV treatment conditions, hypha formation was only observed after 10 to 11 days of incubation (data not shown). These results were confirmed using the plate-washing method (56). After 10 days of growth, the colonies (control and EV treated) were washed using the same flow rate, water temperature, and duration of time (Fig. 8A, bottom panels), producing results that were similar to those obtained under standard conditions.

The capacity to reduce the adhesive and invasive abilities of C. albicans indicated that preincubation of yeast cells with C. albicans EVs could decrease the virulence of C. albicans. To investigate whether the treatment of yeast cells with EVs would impact pathogenesis, we used G. mellonella as a model. Infection of G. mellonella larvae with a lethal inoculum of yeast cells of C. albicans cultivated in standard medium without C. albicans EVs caused melanization and death in 80% of the animals in less than 1 week (Fig. 8B). Remarkably, when the pretreated yeast cells were used to infect the larvae, only 5 to 10% of the animals died (Fig. 8B, experiments 1 and 2). We then evaluated the ability of EVs to impair C. albicans differentiation in the presence of serum, a major hyphae-inducing factor in mammalian hosts (57). Our results demonstrated that C. albicans EVs were able to inhibit yeast-to-hypha differentiation in the presence of serum and CO_2_ (Fig. 8C). Together, these data suggested that C. albicans EVs could directly affect the pathogenesis of C. albicans during the infection.

## DISCUSSION

Our results demonstrated that the addition of C. albicans 90028 EVs strongly inhibited biofilm development of C. albicans. In addition, their filamenting-suppressing activity was similar to the effect of FOH. The effect was notably prolonged, indicating that yeast-to-hyphae morphogenesis was temporarily turned off. The exact mechanism and signaling pathways triggered by C. albicans EVs that promoted the long-lasting effect have not been investigated here, but the process could involve epigenetic changes, as reported previously for the white-opaque transition (58). In addition to epigenetic modifications, other changes triggered by C. albicans 90028 EVs could also be expected and should also be further investigated. For instance, C. albicans EV-treated cells lost their ability to adhere to the plates’ wells after 7 h of incubation, suggesting that treatment with C. albicans EVs caused alterations on the fungal surface composition. Although the activity of C. albicans 90028 EVs was dose dependent, the inhibitory effect was apparently not linked to yeast density in fungal cultures. These data suggest that the C. albicans 90028 EV components involved with morphogenesis must reach a threshold concentration to inhibit differentiation, a circumstance that is well-known for the QSM FOH and tyrosol (15, 17). This hypothesis agrees with the ordinary physiological transition between yeast and hyphal forms, which would not occur regularly if EVs or other extracellular regulators were inhibitory at minimum concentrations. Altogether, these results indicate that C. albicans EVs carry a group of terpenes and fatty acids able to regulate growth and morphogenesis in C. albicans. A recent work published by Bitencourt and colleagues (59) also suggested that fungal EVs could be involved in fungal intraspecies communication and could regulate C. albicans dimorphism. However, in contrast with our results, those authors demonstrated that C. albicans EVs produced by yeasts induced filamentation, although EVs from hyphae exhibited a more prominent effect. In addition, their experiments also suggested that EVs stimulated C. albicans growth, a result not exploited but consistently observed in our current work. These differences might have resulted from the use by Bitencourt et al. (59) of a distinct C. albicans strain (ATCC 64548) and a different culture condition (YPD medium), which could modify the EV composition, as previously reported (60). Moreover, their experimental conditions were limited to 4 h of filamentation, when the morphological impact of EVs was just initiating in our experiments. These contrasting effects reinforce the relevance of further investigating EVs in fungal physiology.

To confirm that C. albicans uses EV export as a general mechanism to control yeast growth and morphogenesis *in vitro*, we isolated C. albicans EVs from two other C. albicans strains with distinct filamentation abilities and virulence profiles and tested their capacity to inhibit yeast-to-hyphae differentiation. Our data revealed that these three different strains of C. albicans produced EVs with similar properties, which were also comparable to other fungal EVs, as recently characterized by Reis and colleagues using similar detection approaches with C. gattii (46). In addition, small differences in EVs size were observed when the distinct strains were compared, confirming previous data published by our group showing that C. albicans strains produced EVs with distinct dimensions (31). Morphological differences of EVs are usually attributed to changes in composition and/or biogenesis pathways. EVs released by the strain 90028 were able to inhibit filamentation of all tested C. albicans strains. Furthermore, C. albicans EVs from different strains also exhibited the ability to block filamentation of C. albicans strain 10231. Thus, the C. albicans EVs’ capacity of inhibiting yeast-to-hypha differentiation appears to be a conserved mechanism.

A number of compounds carried by fungal EVs could be involved with growth and morphogenesis control, including lipids, proteins, and RNA. The participation of thermolabile molecules was discarded since the filamentation inhibition is observed even when the C. albicans EVs are heated. Since lipids are thermostable, these results suggest that a lipid compound could be responsible by the C. albicans EV-mediated inhibitory activity. Given that FOH is a lipid that regulates morphogenesis in C. albicans, we speculate that this molecule could be the component mediating the yeast-to-hypha inhibitory effect. Nevertheless, FOH is not produced by the C. albicans strain 10231 and has never been characterized in H. capsulatum. Our TLC results confirm the presence of an FOH band with inhibitory activity in C. albicans strain 90028, but not in H. capsulatum or S. cerevisiae lipid extracts. However, it is possible that the colorimetric method used in our experiments was not sufficiently sensitive to detect small amounts of FOH. In addition, it was still necessary to confirm that FOH is addressed to C. albicans EVs at appropriate concentrations to inhibit filamentation.

Our GC-MS analysis strongly indicates the presence of lipids in C. albicans EVs that could interfere with morphogenesis, including a family of terpenes and medium-chain fatty acids (17, 22). Besides FOH, other sesquiterpenes and diterpenes are major compounds characterized in the C. albicans 90028 EVs. Of note, the proportion of terpene species in C. albicans EVs is distinct from the LP and displays the largest amounts of FOH isomers. In addition, no FOH has been found in EVs released by the C. albicans strain 10231, S. cerevisiae, and H. capsulatum, which may explain why they have a distinct kinetic to inhibit differentiation compared to EVs released by C. albicans strain 90028.

In previous studies, the inhibitory concentration of FOH affecting C. albicans differentiation *in vitro* was shown to be in the range of 1 to 150 μM (19, 21, 53–55, 61). According to our quantitative analysis, the sterol/protein weight ratio was 1:20 (data not shown). Most of our experiments were developed using a sterol concentration of 5 μg/mL of EV suspension, corresponding to a protein concentration of 100 μg/mL. Considering the lowest concentration of FOH used in previous studies (1 μM), the amount of purified FOH required for yeast-to-hyphae inhibition was 222.3 ng/mL. As demonstrated in Table 2, an EV suspension able to inhibit filamentation contained approximately 11.3 ng/mL of FOH, corresponding to the sum of both Z,E-FOH and E,E-FOH isomers. Thus, it was expected that FOH alone was not fully responsible for the effect exhibited by C. albicans EVs. Supporting this hypothesis, C. albicans 10231 EVs retained the ability to inhibit filamentation despite the absence of FOH. These results also suggest that other lipids besides FOH might mediate the filamentation inhibition in C. albicans, a premise also supported by the fact that FOH has not been found in EVs from H. capsulatum and S. cerevisiae. DHFOH, geranylgeraniol, and dihydrogeranylgeraniol were the additional terpenes detected in high concentrations in C. albicans 90228 EVs. The fact that DHFOH was the only terpene exhibiting a similar activity to FOH indicates that this molecule may contribute significantly to the inhibitory effect mediated by EVs released from C. albicans strain 90028. The biological activity of DHFOH has been previously reported in *in vitro* studies that demonstrated that volatile compounds produced by C. albicans, including DHFOH, inhibited the growth of dermatophytes (62). Taken together, our and previous functional and analytical data suggest that a combination of lipid species could mediate the inhibitory activity in C. albicans.

As mentioned above, our GC-MS data also revealed the presence of different fatty acids species in fungal EVs, including C_12:0_, C_14:0_, C_16:2_, C_18:0_, and C_18:1_. Earlier studies have demonstrated that long-chain fatty acids have no effect on C. albicans differentiation, including octadecanoic and octadecenoic acids (24), major components in C. albicans EVs. Nevertheless, butyric acid, a short-chain fatty acid generated by lactic acid-producing bacteria, inhibits C. albicans germination by 40 and 98% when used at 25 and 100 mM, respectively. Recent studies suggest that the biological activity of fatty acids in C. albicans could be related to their chain length. Murzyn and colleagues (63) have shown that purified C_10:0_ inhibited C. albicans filamentation and reduced its capacity to adhere and form biofilm. Willems and colleagues (64) have confirmed the inhibitory activities of C_10:0_ incorporated in lipid emulsions. Notably, in both studies, C_10:0_ did not suppress yeast growth. The effect of C_14:0_ on C. albicans filamentation, biofilm formation, and virulence has recently been investigated by Prasath and colleagues (23). According to these authors, C_14:0_ blocked yeast-to-hypha differentiation and biofilm formation without compromising fungal viability. In addition, treatment with this fatty acid impacted ergosterol synthesis, sphingolipid metabolism, multidrug resistance, and oxidative stress. The effect of medium-chain fatty acids over C. albicans was investigated by Lee and colleagues (22), and they showed that C_7:0_, C_8:0_, C_9:0_, C_10:0_, C_11:0_, and C_12:0_ negatively affected biofilm formation and filamentation in C. albicans. Remarkably, C_14:0_ was enriched in fungal EVs released by strain 10231, and C_12:0_ was found in high concentrations in EVs from S. cerevisiae and H. capsulatum, suggesting that the inhibitory effect mediated by these EVs was at least in part due to these lipids. Moreover, treatment with C_7:0_ and C_9:0_ modified the expression of biofilm and hyphal growth-regulating genes in a similar fashion to FOH, suggesting that they share the same regulatory mechanism. However, in the same study, the authors stated that cAMP did not reverse the inhibitory effect of fatty acids, which indicated that other signaling pathways related to hyphal development could be impacted. Our experiments showed that if C. albicans yeasts were treated with C. albicans 90028 EVs and then db-cAMP was added to the wells, a significant reversion of effect was observed. However, the ability of db-cAMP to reverse the EVs effect was reduced when db-cAMP and EVs were preincubated before addition to the culture. These results strongly indicate that EVs are able to inactivate the db-cAMP. Although this last condition is not expected to occur *in vivo*, it shows that fungal EVs have different ways to impact the extracellular environment.

It has been speculated that the reduced size and round shape of yeast cells make them the putative forms used by C. albicans to disseminate in the host. However, this possibility is questionable due to the presence of serum in the blood, which would promptly induce germ tube and hypha formation (57). Our experiments showed that C. albicans EVs inhibited yeast-to-hypha transition even in the presence of fetal bovine serum (FBS) and 5% CO_2_ atmosphere, indicating that morphogenesis could be controlled by EVs during various phases of infection. This possibility was corroborated by the long-term effect manifested by C. albicans EVs *in vitro*. Thus, C. albicans EVs would make hypha-to-yeast differentiation feasible *in vivo*, which could possibly explain how this fungus modulates differentiation to facilitate dissemination.

Based on the results discussed above, we have hypothesized that pretreatment of yeast cells *in vitro* with EVs would also decrease C. albican’s ability to invade host cells and cause disease. This possibility is reinforced by the fact that there was a delay during filamentation in the edge of colonies formed by C. albicans EV-treated yeast cells during agar-penetrating growth tests. To correlate the inhibitory effect of C. albicans EVs with the decrease in fungal invasion and virulence, we have investigated the ability of pretreated yeast cells to kill G. mellonella larvae. Indeed, C. albicans EV treatment significantly attenuated the ability of C. albicans to cause larvae death. Melanization was only observed in larvae infected with untreated yeasts, confirming stress due to invasive candidiasis. This result indicated that by inhibiting yeast-to-hypha differentiation, C. albicans EVs decreased fungal virulence. Similar results have been observed when C. albicans was treated with the middle-chain fatty acid C_9:0_ using Caenorhabditis elegans as a model (23). In addition, this validates the idea that controlling filamentation would be a potential approach as a therapeutic strategy (65).

In conclusion, our results support recent findings showing that fungal EVs can be used as messaging compartments and directly influence biofilm production and morphogenesis. In addition, our study presents the fine structural characterization of previously unknown lipid components. EVs could stabilize the lipids and reduce their susceptibility to being oxidized, a function already suggested for liposomes. We have characterized the major players mediating C. albicans yeast-to-hypha differentiation, and further studies are necessary to determine whether these identified lipids in combination could be used to control candidiasis *in vivo*.

## MATERIALS AND METHODS

### Microorganisms and culture conditions.

C. albicans ATCC strains 90028, 10231, and SC5314 and the S. cerevisiae strain RSY225 were cultivated in Sabouraud at 30°C under agitation (150 rpm) for 48 h. H. capsulatum strain G-217B (ATCC 26032) was cultivated in *Histoplasma*-macrophage medium (HMM) at 37°C under agitation (180 rpm). C. albicans yeast cells were washed with phosphate-buffered saline (PBS) 3 times, enumerated, and then transferred to medium 199 (M199), pH 4.0 or pH 7.0, RPMI-MOPS (morpholinepropanesulfonic acid) 0.165 M, or RPMI supplemented with 10% fetal bovine serum (FBS) according to the experiments described below.

### Isolation and quantification of EVs.

EVs were isolated following protocols developed by our group (31) with minor adjustments. Briefly, yeast cultures (1 L) from each fungal species were collected after 48 h of growth as described above. Cells and debris were removed by sequential centrifugation steps (4,000 and 15,000 × *g*, 15 min, 4°C). An additional step of filtration using a 0.45-μm membrane filter (Merck Millipore) was used to remove any remaining yeast cells or debris. The supernatant was then concentrated using an Amicon ultrafiltration system (100-kDa cutoff; Millipore) to a final volume of approximately 20 mL. EVs were collected by centrifugation at 100,000 × *g* for 1 h at 4°C. Supernatant was discarded, and the pellet enriched in EVs was washed twice with PBS, pH 7.4, at 100,000 × *g* for 1 h at 4°C.

Alternatively, 300 μL containing 10^7^ C. albicans yeast cells was plated onto petri dishes containing Sabouraud dextrose agar (SDA). After 24 h of growth, yeast cells were harvested using a cell scraper and transferred to 20 mL of PBS. Cells and debris were removed as described above, the supernatant was filtered using a 0.45-μm membrane filter, and the EVs were collected by centrifugation at 100,000 × *g* for 1 h at 4°C. To isolate EVs from macrophages (RAW 264.7 macrophage-like; ATCC TIB-71) culture medium RPMI supplemented with 10% FBS (400 mL), FBS was ultracentrifuged to remove vesicles present, with 70% cellular confluence, and was collected after 24 h and centrifuged (300 × *g* and 15,000 × *g*, 15 min, 4°C). The same steps of filtration were used, and the EVs were recovered after centrifugation at 100,000 × *g* for 1 h at 4°C.

The presence of EVs was confirmed using dynamic light scattering (DLS) analysis (31) and nanoparticle tracking analysis (NTA) (46). EVs were suspended in 200 μL PBS, and aliquots of 5 to 10 μL were used to perform the sterol quantification. Sterol content was determined using the quantitative fluorometric kit Amplex red sterol assay kit (Molecular Probes, Life Technologies). Protein content was measured using the bicinchoninic acid (BCA) kit (Pierce, Thermo Fisher Scientific). Additionally, aliquots of 5 to 10 μL were also plated onto Sabouraud agar plates to confirm that the preparation was sterile.

### NTA of C. albicans EVs.

Nanoparticle tracking analysis (NTA) of fungal EVs was performed on an LM10 nanoparticle analysis system, coupled with a 488-nm laser and equipped with a scientific complementary metal oxide semiconductor (SCMOS) camera and a syringe pump (Malvern Panalytical, Malvern, UK) following the conditions used by Reis and colleagues (46). All samples were 833- to 2,500-fold diluted in filtered PBS and measured within the optimal dilution range of 9 × 10^7^ to 2.9 × 10^9^ particles/mL. Samples were injected using a syringe pump speed of 50, and three videos of 60 s were captured per sample, with the camera level set to 15, gain set to 3, and viscosity set to that of water (0.954 to 0.955 cP). For data analysis, the gain was set to 10 to 15, and the detection threshold was set to 2 to 3 for all samples. Levels of blur and maximum jump distance were automatically set. The data were acquired and analyzed using the NTA 3.0 software (Malvern Panalytical).

### Biofilm assays.

For biofilm production and quantification, two protocols were used. First, yeast cells were plated onto polystyrene 96-well plates containing M199 (10^5^ cells/well) in the presence of different concentrations of EVs (0.5, 1, and 5 μg/mL based on sterol content). Second, yeasts were plated onto polystyrene 96-well plates containing M199 (10^5^ cells/well), and the systems were incubated for 90 min. Then, the nonadherent cells were removed by washing with PBS, and fresh M199 was added containing different concentrations of EVs (0.5, 1, and 5 μg/mL based on sterol content). For both protocols, PBS was used as negative control. Biofilm was allowed to develop for 24 and 48 h at 37°C and then quantified by the crystal violet method. Briefly, after each time point, the biofilm-coated wells were washed 3 times with PBS, air-dried for 45 min, and stained with 0.4% aqueous crystal violet (100 μL) for 10 min and washed 3 times with 200 μL of PBS. Then, 100 μL of absolute ethanol was added to the wells, and the systems were incubated for 5 min. The plate was then analyzed using a microplate reader (EL808; BioTek) at the wavelength of 540 nm (66). Tests were performed in triplicate. For scanning electron microscopy, yeast cells were washed three times in PBS, pH 7.2 ± 0.1, to remove planktonic cells and fixed in 2.5% glutaraldehyde type I in 0.1 M sodium cacodylate buffer (pH 7.2 ± 0.1) for 1 h at room temperature. After fixation, cells were washed in 0.1 M sodium cacodylate buffer (pH 7.2 ± 0.1) containing 0.2 M sucrose and 2 mM MgCl_2_. Then, cells were dehydrated in ethanol (30, 50, and 70%, for 5 min and then 95% and 100% twice for 10 min), subjected to critical point drying in an EM CPD300 (Leica, Wetzlar, Germany), covered with gold sputtering (10 ± 2 nm), and visualized in a Zeiss Auriga 40 (Zeiss, Oberkochen, Germany) microscope operated at 2 kV.

### Yeast-to-hyphae differentiation.

C. albicans yeast cells (2.5 × 10^3^/well) were plated onto 96-well plates containing the hypha-inducing medium M199 or RPMI supplemented with 10% FBS. To test whether the number of yeast cells would influence the EV activity, different suspensions of C. albicans were used (1.5 × 10^3^, 2 × 10^4^, and 3.5 × 10^4^ yeast cells/well). Fungal EVs were added at final concentrations varying from 0.025 to 5 μg/mL (based on sterol content). For the lipid extracts, activity tests aliquots of 10 μL (equivalent to 5 μg/mL of EVs) were added to the wells. The purified terpenes DHFOH (Penta, USA) and geranylgeraniol (Sigma-Aldrich, USA), solubilized in 1 mL of ethanol, and ascorbic acid 2,6-dihexadecanoate (TCI America, USA), dissolved in 1 mL of acetone, were added to the wells in different concentrations (please see the figure legends). Plates were incubated at 37°C and 5% CO_2_ for 4, 8, and 24 h. For each time interval, the cells’ morphologies were visualized under an Observer Z1 microscope (Carl Zeiss International, Germany), and the images were collected and processed with ImageJ (NIH, US) and Photoshop (Adobe). Time-lapse video microscopies were performed after plating yeast cells of C. albicans strain 90028 (5 × 10^3^ yeast cells/well) onto 4-well plates containing 1 mL of M199 medium. A final concentration of 5 μg/mL of C. albicans EVs was added to the wells. PBS was used as control. Samples were placed in a culture chamber with controlled temperature and CO_2_ environment (37°C and 5%, respectively) and attached to a Nikon Eclipse TE300 (Nikon, USA). Phase-contrast images were captured every minute using a Hamamatsu C2400 charge-coupled-device (CCD) camera (Hamamatsu, Japan) connected with a Scion FG7 frame grabber (Scion Corporation, USA). Time-lapse images were taken every 1 min using a Nikon Eclipse TE300 microscope attached to a digital Hamamatsu C11440-10C camera (Hamamatsu, Japan). Movie animations were generated using ImageJ v1.8 software (NIH, US).

### EV lipid extraction.

An aliquot containing 50 μg/mL of EVs was dried using a SpeedVac vacuum concentrator (Eppendorf), followed by lipid extraction with chloroform (C)/methanol (M)/water (W) (8:4:3 [vol/vol/vol]) according to Nimrichter et al. (67). The pellet containing nonextractable residues and precipitated protein was recovered by centrifugation. Partition was obtained after water addition to the system to reach the proportion C/M/W (8:4:5.6 [vol/vol/vol]). The upper and lower phases and the protein content were dried separately using SpeedVac, suspended in dimethyl sulfoxide (DMSO), and their activity tested as described below. The lower (LPs) and upper phases (UPs) contain lipids with higher and lower polarity, respectively. The interphase was also dried using SpeedVac and suspended in DMSO. Aliquots of each fraction (pellet and lower and upper phases) equivalent to 5 μg/mL (based on sterol content) of EVs according to previous quantification were tested. The same protocol was used to separate the nonpolar components of Sabouraud medium, and the lower phase was used as control.

### Thermostability of EV activity.

To investigate the thermostability of the C. albicans 90028 yeast EV activity, the samples were heated for 15 min at 90°C. Then, a final concentration corresponding to 5 μg/mL (based on sterol content before heat treatment) of EVs was tested as described above.

### Long-term effect of EVs.

C. albicans yeast cells (2.5 × 10^3^/well) were plated onto 96-well plates containing M199 and treated with 5 μg/mL (based on sterol content) of EVs. The cell morphology of C. albicans was analyzed using an Observer Z1 microscope (Carl Zeiss International, Germany) after 4, 8, and 24 h. The yeast cells were collected after 24 h of incubation in the presence of EVs and washed three times with PBS. Yeast cells were enumerated, and a total of 2.5 × 10^3^/well were transferred to fresh M199 in the absence of EVs. These steps were repeated four times without addition of EVs to the cultures. Untreated yeast cells cultivated in M199 were used as control every 24 h. These steps were repeated four times.

### Hyphae-to-yeast differentiation.

Yeast cells of C. albicans (2.5 × 10^3^/well) were plated onto 96-well plates containing M199 media and then incubated for 4 h to allow yeast-to-hypha differentiation. After this period, 100% of the yeast cells differentiated to hyphae. Then, a final concentration of EVs (5 μg/mL, based on sterol content), EVs (5 μg/mL), FOH (80, 8 and 0.8 μM), and DHFOH (80, 8 and 0.8 μM) were added to the wells and the morphology accompanied for additional 4, 8, and 24 h as described above. PBS was used as control. Tyrosol stock was diluted in methanol.

### db-cAMP effect on EV inhibitory activity.

Yeast cells of C. albicans (2.5 × 10^3^/well) were plated onto 96-well plates containing M199 and treated with 5 μg/mL (based on sterol content) of C. albicans 90028 EVs preincubated with the cAMP donor db-cAMP (10 mM) (Sigma-Aldrich) for 1 h. Alternatively, yeasts were treated with C. albicans 90028 EVs for 1 h, and then db-cAMP (10 mM) was added to the wells. Cell morphology was analyzed using an Observer Z1 microscope (Carl Zeiss International, Germany) after 4, 8, and 24 h.

### Yeast lipid extraction.

Yeast of C. albicans, H. capsulatum, and S. cerevisiae (6 × 10^10^ cells) was suspended in a mixture of C/M (2:1 [vol/vol]), stirred for 16 h at room temperature, and then clarified by centrifugation. Cells were extracted with a mixture of C and M (1:2 [vol/vol]) under the same conditions. Lipid extracts were combined and dried using a rotavapor (Heidolph, Germany). The lipids were then suspended in a mixture of C/M/W (8:4:5.6 [vol/vol]) and vigorously mixed. The lower phase was dried using a rotavapor and suspended in 2.5 mL of chloroform. Aliquots were dried using Vacufuge Plus vacuum concentrator (Eppendorf, Germany) and suspended in DMSO. A yeast-to-hypha differentiation assay was performed to determine the minimal amount of the lower phase from C. albicans (20, 4, 2, 1, 0.5, 0.25, 0.125, and 0.05 mg/mL) was able to block the differentiation.

### Size exclusion chromatography.

To exclude the presence of free-contaminants in EV preparations, we used size exclusion chromatography (SEC) according to Menezes-Neto and colleagues (68) with minor adjustments. Briefly, Sepharose 4B (final volume of 10 mL) was packed in a 10-mL syringe and then equilibrated with PBS-citrate 0.36% (wt/vol). The EVs obtained after ultracentrifugation were suspended in a final volume of 500 μL and applied to the column. A total of 30 fractions of 500 μL were collected immediately after column loading using PBS-citrate as eluant. EVs aliquots of 5 to 10 μL were used to perform the sterol and protein quantification as described previously. A yeast-to-hypha differentiation assay was performed to determine the activity of each fraction. To avoid additional steps to concentrate the samples, the lipids from each fraction were extracted as described above and the LP normalized according to the sterol content. A final concentration of 5 μg/mL of sterol was used. For the fractions where sterol was not detected, 400 μL of each fraction was used for lipid extraction and solvent partition. The LP was dried down in a SpeedVac and suspended in 2 μL of DMSO. An aliquot of 1 μL was used to test the inhibitory activity.

### Reverse-phase thin-layer chromatography.

EVs or LP lipids were suspended in C/M (1:1 [vol/vol]), and their concentration was normalized by sterol content or the total weight, respectively. Aliquots of 10 μL were spotted into reverse-phase TLC (RP-TLC) plates (silica gel 60 RP-18 F254s; 1 mm; Merck, Germany). The plates were developed in chambers presaturated for 10 min using acetone as a solvent system. After development, plates were dried at room temperature, and the bands were visualized after spraying with a solution of 50 mg ferric chloride (FeCl_3_) in a mixture of 90 mL water, 5 mL acetic acid, and 5 mL sulfuric acid (69). After spraying, plates were heated at 100°C for 3 to 5 min. FOH was used as standard. Under these conditions, FOH appears as a purple band. Alternatively, the developed plates were incubated in a saturated iodine chamber where the lipids appear as yellow bands. The bands were scraped from the TLC and placed into microcentrifuge tubes. Lipids were suspended in C/M (1:1 [vol/vol]) and filtered using a polyvinylidene difluoride (PVDF) membrane with 0.22 μm to a new microcentrifuge tube. Samples were dried down using a SpeedVac, suspended in DMSO, and normalized by lipid weight. For the lipid activity tests, aliquots of 10 μL (equivalent to 5 μg/mL of EVs) were added to the wells containing yeasts of C. albicans in M199 medium. Plates were incubated at 37°C and 5% CO_2_ for 4, 8, and 24 h. For each time point, the cells’ morphologies were visualized under an Observer Z1 microscope (Carl Zeiss International, Germany), and the images were collected and processed with ImageJ (NIH, USA) and Photoshop (Adobe).

### FOH extraction.

The C. albicans yeast 90028 and 10231 EV samples (19.1 μg total sterol) were resuspended in 400 μL methanol (high-performance liquid chromatography [HPLC] grade; Fisher Scientific), vortexed for 2 min, and centrifuged for 15 min at 14,000 × *g* at room temperature. The supernatants were transferred to a 2-mL polytetrafluoroethylene (PTFE)-lined cap glass tube (Supelco; catalog no. 27134; Sigma-Aldrich). The ensuing pellet was redissolved in 400 μL methanol, and the previous steps were repeated. The two resulting supernatants were pooled and dried under nitrogen gas in Supelco PTFE-lined cap glass vials. For FOH extraction, the yeast C. albicans yeast 90028 and 10231 EV samples were partitioned by adding 500 μL HPLC-grade water (Fisher Scientific) and 500 μL ethyl acetate (Sigma-Aldrich; catalog no. 270989) and vortexed for 2 min. After phase separation, the upper (organic) phase was collected, transferred to a fresh PTFE-lined cap glass vial, and dried under nitrogen gas. Samples were then redissolved in 20 μL methylene chloride (Optima grade, Fisher Scientific), transferred to Verex PTFE-lined/silicone cap vials (Phenomenex; μVial i3 [Qsert]) just prior to gas chromatography-mass spectrometry (GC-MS) analysis.

### Lipid analysis by GC-MS.

A standard of FOH isomer mixture (catalog no. 93547; Supelco, Sigma-Aldrich) was diluted in methylene chloride (Optima grade, Fisher Scientific) to a concentration of 22.5 pmol/μL to determine the transition ions to be used in the selected-reaction monitoring (SRM) method. Chromatographic separation was performed by injecting 1 μL of FOH standard onto a Thermo TSQ 9000 triple-quadrupole GC-MS/MS system (Thermo Fisher Scientific) set to splitless, MS transfer line at 250°C, with an AI/AS 1310 series autosampler (Thermo Fisher Scientific), equipped with a Zebron (ZB-FFAP; 30-m by 0.32-mm by 0.25-μm) column, previously indicated for separation of FOH isomers (51). Isomers were separated by maintaining a loading temperature of 40°C for 1.2 min, followed by a heat gradient to 250°C at a rate of 20°C/min, and a plateau held at 250°C until the end of the run at 13.5 min. Additionally, ramped pressure was applied throughout the run, beginning with 89 kPa held for 2 min, and increased to 150 kPa over 6.1 min, while holding at 150 kPa until the end of the run. Initially, a full scan was run to determine the retention time (RT) and underivatized FOH limits of detection prior to FOH SRM confirmation and full scan analysis.

The lowest detectable level of underivatized FOH isomers from the standard, using the automated SRM (AutoSRM) mode on the TSQ 9000 triple-quadrupole GC-MS (Thermo Fisher Scientific) with an electron ionization (EI) source temperature of 320°C, was 4.5 fmol/μL. However, subsequent analyses were performed with a concentration of FOH standard of 4.5 pmol/μL, in which the detection of all four isomers (Z,Z, Z,E, E,Z, and E,E) of FOH was reproducible, and the signal of the least abundant isomer (Z,Z) was at least ∼10% of the most abundant (E,E) isomer and ∼6-fold higher than the background. Using the AutoSRM mode on the TSQ 9000 GC-MS dashboard feature, the ion fragmentation was optimized at their respective RT with the top three transition ions (81.1 to 79.1 at 8 collision energy [CE], 69.1 to 41 at 6 CE, and 41.1 to 39.1 at 8 CE) were chosen and imported into Chromeleon v7.2.9 software. Images of the SRM chromatograms of the FOH standard and the samples extracted from C. albicans yeast 90028 and C. albicans 10231 EVs were transferred from Chromeleon v7.2.9 into PowerPoint for figure generation.

### Quantitative analysis of FOH or FOH-like molecules by GC-MS.

FOH quantitation was performed by building a standard curve with d6-E,E-FOH (catalog no. SKU700294O; Avanti Polar Lipids, Alabaster, AL) at concentrations of 0.1, 1, 2.5, and 5 ng/μL. Peak areas of the standard and samples were imported into GraphPad Prism 9.0.0 for formula and *R*^2^ generation and graph.

### Agar invasion assay.

Hyphal growth was stimulated using M199 medium. C. albicans (90028) yeast cells were treated or not with EVs (5 μg/mL, based on sterol content) overnight, and a suspension of yeast cells 10 μL containing 10^5^ cells was plated onto M199 agar plates. Plates were then incubated at 37°C, and growth was monitored daily for hypha formation. After 10 days of incubation, colonies were washed in a stream of water using the same flow rate, water temperature, and duration of time as described by Cullen (56). Colony images were taken with an Optiphase microscope coupled with a digital camera (AmScope) and processed using Adobe Photoshop.

### Galleria mellonella infection.

Larvae of wax moth G. mellonella were used to investigate virulence of EV-treated yeast cells (70). C. albicans yeast cells (2.5 × 10^3^) were treated with EVs (5 μg/mL, based on sterol content) in M199 for 24 h. Yeast cells were then washed in PBS and enumerated. As a control, yeast cells cultivated in Sabouraud were used. A final volume of 10 μL containing 2 × 10^5^ yeast cells was injected into the last right proleg of the larvae (200 to 300 mg each) using a Hamilton syringe. An additional control included larvae injected with 10 μL PBS alone. The injected larvae were kept at 37°C, and the number of deceased subjects per group was monitored daily.

### Statistical analysis.

All statistical analyses were performed using GraphPad Prism 6 v5.02 for Windows (GraphPad Software).

Supplemental material can be found at 10.6084/m9.figshare.19403894.
